# Digital Technology for Internet Access by Patients With Early-Stage Schizophrenia in Spain: Multicenter Research Study

**DOI:** 10.2196/11824

**Published:** 2019-04-05

**Authors:** Patricia Fernández-Sotos, Antonio Fernández-Caballero, Pascual González, Ana Isabel Aparicio, Isabel Martínez-Gras, Iosune Torio, Mónica Dompablo, Lorena García-Fernández, José Luis Santos, Roberto Rodriguez-Jimenez

**Affiliations:** 1 Department of Psychiatry Instituto de Investigación Sanitaria Hospital 12 de Octubre (imas12) Madrid Spain; 2 CIBERSAM (Biomedical Research Networking Centre in Mental Health) Madrid Spain; 3 Instituto de Investigación en Informática Albacete Spain; 4 Departamento de Sistemas Informáticos Universidad de Castilla-La Mancha Albacete Spain; 5 Servicio de Psiquiatría del “Hospital Virgen de la Luz” Cuenca Spain; 6 Universidad Rey Juan Carlos Madrid Spain; 7 Hospital Universitario San Juan Alicante Spain; 8 CogPsy-Group Universidad Complutense de Madrid Madrid Spain

**Keywords:** information technology, computers, internet, schizophrenia

## Abstract

**Background:**

Digital technology and social networks are part of everyday life in the current internet age, especially among young people. To date, few studies have been published worldwide on the pattern of use of digital technology devices and applications in patients with early-stage schizophrenia and even fewer comparing them with healthy participants (not using data from general population surveys) from the same demographic areas. In Spain, no such study has been carried out.

**Objective:**

The aim of this study was to analyze how patients with early-stage schizophrenia use internet and social networks compared with healthy participants matched by age and gender and also to examine which devices are utilized to access internet resources.

**Methods:**

A cross-sectional, multicentric study was carried out through a semistructured interview asking about the use of digital technology devices and internet. The sample comprised 90 patients and 90 healthy participants. The semistructured interview was conducted on 30 outpatients and 30 healthy subjects in each of the 3 different cities (Madrid, Alicante, and Cuenca). Student *t* test was used for continuous variables and chi-square test for categorical variables. In the case of ordinal variables, nonparametric Mann-Whitney *U* and Kruskal-Wallis *H* tests for independent samples were performed to compare groups.

**Results:**

The results indicated that a large proportion of patients with early-stage schizophrenia have access to different digital devices and use them frequently. In addition, both groups coincide in the order of preference and the purpose for which they use the devices. However, a lower frequency of use of most digital technology devices was detected in patients compared with healthy participants. In the case of some devices, this was due to the impossibility of access and not a lack of interest.

**Conclusions:**

To our knowledge, this is the first study to analyze patterns of internet access and use of digital technology devices and applications in Spanish patients with early-stage schizophrenia compared with healthy participants from the same demographic areas. The results on significant access and use of digital technology and internet shown in this cross-sectional study will allow enhanced and more efficient treatment strategies to be planned, utilizing digital technology devices, for patients with early-stage schizophrenia.

## Introduction

### Digital Technology

As indicated in a very recent report by the International Telecommunication Union (ITU) and the United Nations Educational, Scientific and Cultural Organization, the growth in information and communication technologies and the massive use of internet in the last 20 years have influenced the development of human activity in multiple areas such as education and health [1]. In this sense, internet access has increased worldwide over the last 10 years, reaching 48% of the population in 2017; in the case of Europe, the ITU reports a statistic of 79.6%. Facebook seems to be the favorite application throughout the world, surpassing 2 billion active users per month and reaching 1320 million active users per day in June 2017 (of whom, approximately 91% access Facebook via mobile technologies). WhatsApp and YouTube follow closely with around 1 billion users in June 2017 [1].

Moreover, in 2017, according to the Instituto Nacional de Estadística (INE; Spanish National Institute of Statistics), 78.4% of households with at least one member, aged between 16 and 74 years, had a computer and 97.4% of households had a landline or mobile phone [2]. In addition, 8 out of 10 participants between the ages of 16 and 74 years had used the internet in the previous 3 months and 2 out of every 3 had done so daily, with the use being even more frequent among young people. In the previous 3 months, 98% of young people aged between 16 and 24 years had used the internet, 91.3% on a daily basis and 49% for Web-based purchase. The products and services most commonly acquired were holiday accommodation (54.1%), sports equipment, clothing (53.5%), tickets for shows (47.6%), and other services for trips (44.7%). When analyzing the types of activities carried out on the internet by the Spanish population in 2017, those that were most frequently performed were receiving or sending emails; reading news, newspapers, or Web-based news magazines; searching for information about services; looking for information about health issues; and participating in social networks [2].

According to the same statistics by the INE, the device most commonly used to connect to the internet was by far the smartphone (90.4% of internet users in the last 3 months), followed by laptop (39.3%), desktop computer, and tablet. Smart television (TV), other mobile devices, and game consoles were also mentioned (12%). Regarding participation in social networks, during the previous 3 months, 67.6% of internet users had participated in general nature social networks such as Facebook or Twitter, creating a user profile or sending messages and other contributions, with greater participation by women than by men. The most participative subjects were students (90.4%) and people aged between 16 and 24 years (90.0%).

The fact that technology is rapidly changing society, and many activities now require the ability to use digital technology, potentially poses new problems for several population groups, including older adults, the economically disadvantaged, and people with severe mental illness [3]. Moreover, despite the clear potential of digital technology to connect people and health data in new ways, a key challenge is to ensure that patients and their needs remain at the center of technology development and implementation [4,5].

### Use of Digital Technology in Health Care

In the last 10 years, the development and use of mobile devices devoted to health has increased significantly. The main advantage of these devices is that they increase access to medical care, reduce costs, and offer new options for control, prevention, detection of diseases, and basic diagnosis [6].

The literature at the time of this study reports positive effects derived from the use of mobile apps for health in relation to improving hygienic dietary habits such as stopping smoking, losing weight, dieting, and physical activity, as well as increasing therapeutic adherence and preventing and treating sexually transmitted diseases [7-9].

### Use of Digital Technology in Mental Health

The impact of psychological interventions on mental disorders is unquestionable. However, owing to the limited resources of medical and psychological care and availability of interventions, their enormous potential is restricted [10-11]. Using internet and mobile-based interventions for mental disorders offer an accessible, innovative, and personalized option that addresses several of the devastating effects of mental illness, including associated stigma and the chronic nature and symptoms of these disorders. Patients are thus empowered to participate actively in their recovery [12]. Although digital exclusion among people with mental illness is still present, especially in older individuals with severe mental disorders, patients’ access to internet-enabled technology is growing [13]. Internet and mobile-based studies or interventions have been shown to be effective in understanding and managing different mental disorders such as substance use disorder [14], depression [15], anxiety [16,17], and schizophrenia [18].

### Use of Digital Technology in Schizophrenia and Related Disorders

Schizophrenia is a chronic psychiatric disorder that affects approximately 1% of the population and severely limits the social and occupational functioning of patients [19]. Symptoms of schizophrenia include positive symptoms (hallucinations, delusions, and disorganization of language and behavior), negative symptoms (including abulia, associability, anhedonia, and alogia or affective flattening), alterations in mood, and deficits in cognition [20].

Schizophrenia is a mental disorder for which mobile health (mHealth) offers a tremendous opportunity to provide personalized, innovative, and accessible solutions. A recent systematic review concluded that internet and mobile-based interventions for psychosis seem to be cost-effective, accessible, acceptable, feasible, and have the potential to improve clinical and social outcomes [21]. A subsequent review including studies carried out in 12 different countries supported the feasibility and acceptability of emerging mHealth and electronic health (eHealth) interventions among people with serious mental illness (including schizophrenia, schizoaffective disorder, and bipolar disorder) [22]. Recently, the need for individuals with schizophrenia to engage themselves in Web-based activities was demonstrated [23].

At the time of this study, there was limited information on the access and use of digital technology by psychotic patients. Several previous studies have investigated the prevalence of use of technological devices and applications through surveys. In the United States, 2 studies were conducted in patients diagnosed with schizophrenia and schizoaffective disorder. The first interviewed 457 participants by means of a Web-based survey [12] and the second surveyed 80 patients in Georgia [24]. In addition, 2 other studies were carried out to compare access and the use of internet in persons with different mental disorders, the first in France [25] and the second in Australia [26]. In Europe, 2 other studies examined access and purpose of internet use in patients with schizophrenia. The first study compared patients from Greece and Finland [27] and the second, patients from 2 psychiatric units in Finland [28].

To our knowledge, only 2 studies have been conducted in patients with early-stage schizophrenia. The first study included 71 patients at different stages of their 5-year treatment in a therapeutic program in Montreal (Canada). The patients completed a survey on their access and use of technology and related activities. The authors concluded that a significant proportion of patients with early-stage schizophrenia had access to and were using different technological devices in their daily lives [29]. The second study, carried out in Valencia (Spain), compared access, use, and interest in new technologies and eHealth interventions through a survey on internet use conducted on 65 patients with early psychosis compared with 40 patients with chronic psychosis [30].

It should be noted that in the previously mentioned publications, the use of digital technology for internet access was compared with data of use from general population surveys, but not with a sample of controls matched in age, gender, and place of residence. The lack of a control group, as well as a nonmulticentric study design, could be an important source of bias in these publications.

### Objectives

To date, no multicentric studies have been carried out that provide information about the use of digital technology to access the internet by patients with early-stage schizophrenia compared with a sample of control participants matched in age, gender, and place of residence. Hence, we conducted a cross-sectional, multicentric study to analyze the use of digital technology in patients with early-stage schizophrenia and to compare the responses with regard to healthy participants matched by age, gender, and place of residence through a semistructured interview. The following 3 main questions were analyzed:

1. Which digital technology devices are most frequently used by patients with early-stage schizophrenia to access the internet? Is there any difference in use by gender, age, education level, or location? And, primarily, is use similar in healthy participants?
2. What is the main purpose of the use of digital technology devices in early-stage schizophrenia patients? Moreover, is there any difference in the purpose of use between patients and healthy participants?
3. Which are the applications more frequently used when patients access the internet? Is their use similar in healthy participants?

## Methods

### Study Design

This was a cross-sectional, multicentric study of 6 months’ duration (June to November 2017). The design included 3 recruitment centers: *Hospital Universitario 12 de Octubre* (Madrid), *Hospital Virgen de la Luz* (Cuenca), and *Hospital Universitario de San Juan* (Alicante). The study was approved by the Clinical Research Ethics Committee.

The psychiatric service of the *Hospital Universitario 12 de Octubre* serves a population of about 450,000 inhabitants and has an integrated psychosis assistance program, including an intensive program for first psychotic episodes. The psychiatric service of the *Hospital Virgen de la Luz* serves a population of 150,000 inhabitants and also offers an integrated psychosis program. Finally, the psychiatric service of the *Hospital de San Juan* serves a population of 300,000 inhabitants and manages a program for first psychotic episodes.

### Participants

The patient sample size was established as 90 patients (30 patients from each of the centers). All met the Diagnostic and Statistical Manual of Mental Disorders, fifth edition, (DSM-5) diagnostic criteria for schizophrenia assessed with the Structured Clinical Interview for DSM-5 [31]. All the patients were at an early stage of the disorder (5 years or less since their first episode). The mean time of evolution of the disorder was 2.6 years (SD 1.3). A total of 90 healthy participants were recruited (30 from each center), matched with patients for age and gender.

**Table 1:** Sociodemographic data of the samples.

Participants		Patients (n=90)	Healthy participants (n=90)	Statistics		
				Student *t* test (df)	Chi-square test	*P* value
Age (years), mean (SD)		28.1 (SD 8.6)	27.9 (SD 8.7)	0.189 (179)	—^a^	0.85
**Gender (%)**						
	Men	53	57	—	0.2	0.65
	Women	47	43	—	0.2	0.65
**Educational level (%)**						
	Basic	29	22	—	8.4	0.02
	Medium	50	37	—	8.4	0.02
	High	21	41	—	8.4	0.02

^a^Not applicable.

### Inclusion and Exclusion Criteria

The following inclusion criteria were established for patients:

1. Meeting DSM-5 diagnostic criteria for schizophrenia.
2. Staying clinically stabilized during the 3 months before the semistructured interview, according to criteria already used by our group [32].
3. Being an outpatient.
4. Being aged between 18 and 55 years.
5. Speaking fluent Spanish.
6. Providing signed, informed consent.

The following exclusion criteria were considered:

1. Other Axis I major mental disorders of DSM-5.
2. Intellectual disability (IQ <70).
3. Suffering somatic pathology that might interfere with accessing the internet.

The patients were screened by their psychiatrist to determine whether they met the inclusion/exclusion criteria and were thus suitable to participate in the study.

Table 1 shows the sociodemographic data of patients and controls. As expected, there were no differences in age or gender; the only differences were in educational level.

### Data Collection Procedure

The patients were recruited consecutively at the clinical appointments of their respective first-episode programs. After the clinical evaluation, the psychiatrist assessed the inclusion and exclusion criteria and proposed their participation in the study on the frequency and purpose of using a series of digital technology devices. Multimedia Appendix 1 includes all the information on the data collection procedure considering the 32-item checklist of the Consolidated Criteria for Reporting Qualitative Research [33]. The healthy participants were recruited from areas of similar sociocultural status as the patients, mainly from similar cultural and social groups.

### Statistical Analysis

Statistical analysis of all data was conducted using SPSS version 23.0 (IBM Corp). The means and SDs were used to describe continuous variables whereas percentages and chi-square tests were used for categorical variables. Student *t* test was used for continuous variables with a normal distribution. For variables found not to be normally distributed after using Levene test (of homogeneity of variance or homoscedasticity) and ordinal variables, nonparametric tests for independent samples were performed.

The tables described in the section on frequency of use include variables that are considered ordinal variables as they are clearly ordered from *never* (0), including both *never, but would like to* and *never, and would not like to*; *rarely* (1); *once a week* (2); *twice a week* (3); and *every day* (4). Therefore, the *P* value of nonparametric tests for independent samples is presented as a result. The Mann-Whitney *U* test (also called Mann-Whitney-Wilcoxon test or Wilcoxon rank sum test) is performed when we have 2 groups of variables whereas the Kruskal-Wallis *H* (Kruskal-Wallis one-way analysis of variance) test is conducted for more than 2 (in our case, always 3) groups of variables.

## Results

The results for the technological devices used by patients and how they used them are presented. We analyze whether there were any differences for several features such as gender, age, education level, and place of residence. In addition, the use of these digital technology devices by patients with early-stage schizophrenia and healthy participants is compared. Subsequently, the purpose of use of these devices is shown, analyzing 4 different domains: entertainment, work, socialization, and shopping. In addition, the websites used to access these internet services and the main applications used for socialization are studied. Finally, the favorite videogames played by both groups are analyzed.

### Frequency of Use of Digital Technology Devices

A first analysis studied the frequency of use by patients with early-stage schizophrenia of several technological devices that are useful for internet access. In this selection, some devices frequently used for this task such as computers or smartphones and other more novel or not so widely used devices (tablet, game console, or smart TV) were included. The inclusion of these latter devices allows possible trends in use to be evaluated. Moreover, this variety of devices was included to cover different domains and usages.

Table 2 shows the frequency of use of these devices by patients with early-stage schizophrenia. In this table, not only the real frequency of use was included, taking values *every day*, *twice a week*, *once a week,* and *rarely*, but also in the case of *never* for a specific device, an analysis of patients’ interest in their use (*never, but would like* and *never, and would not like*) was included. This variable reveals the actual motivation of low use of a specific device.

Considering the results shown in Table 2, the use of *smartphone* clearly prevails over other devices. More than 80% of patients (75 out of 90 participants) use this device *every day* and it is very popular among patients. The other type of device most frequently used is the computer. In this case, use is not so frequent but the general use of these devices by patients is similar to that of smartphones.

In contrast to the devices previously analyzed, the other 3 are used less frequently, but as can be seen in Table 2, patients’ interest in their use is high.

### Comparison Between Patients and Controls

First, we analyzed whether the frequency of use of these devices is similar between schizophrenic patients and healthy participants. As can be seen in Table 3, the frequency of use of most digital technology devices under study is higher in healthy participants than patients.

As can be seen, there is a significant statistical difference in almost all devices. Only the use of *game console* has a *P* value higher than .05.

Table 4 shows that there are no statistically significant differences between female and male patients in the use of digital technology devices. However, in general, females used *smartphone* and *computer* more frequently and, more remarkably, *game console* is used by a proportional number of each gender. In contrast, males were more frequent users of *smart TV* and *tablet* use was similar between genders.

**Table 2:** Frequency of use of digital technology devices in patients with early-stage schizophrenia (N=90).

Device	Every day, n (%)	Twice a week, n (%)	Once a week, n (%)	Rarely, n (%)	Never, but would like to, n (%)	Never, and would not like to, n (%)
Computer	50 (56)	17 (19)	9 (10)	7 (8)	5 (6)	2 (2)
Tablet	8 (9)	2 (2)	8 (9)	17 (19)	33 (37)	22 (24)
Smartphone	75 (83)	9 (10)	0 (0)	0 (0)	4 (4)	2 (2)
Game console	9 (10)	11 (12)	19 (21)	15 (17)	21 (23)	15 (17)
Smart TV	18 (20)	4 (4)	6 (7)	10 (11)	31 (34)	21 (23)

**Table 3:** Comparison between patients with early-stage schizophrenia (N=90) and healthy participants (N=90) using the Mann-Whitney U test on the frequency of use of digital technology devices.

Devices	Patients, mean (SD)	Healthy participants, mean (SD)	*P* value^a^
Computer	3.04 (1.306)	3.72 (0.750)	<.001
Tablet	0.79 (1.250)	1.22 (1.322)	.006
Smartphone	3.63 (1.022)	3.96 (0.422)	<.001
Game console	1.36 (1.376)	1.63 (1.532)	.23
Smart TV	1.18 (1.619)	1.97 (1.757)	.002

^a^Significance *P*<.05*.*

**Table 4:** Use of digital technology devices in patients with early-stage schizophrenia divided by gender using the Mann-Whitney *U* test (N=90).

Digital device	Gender		*P* value^a^
	Female (47%), mean (SD)	Male (53%), mean (SD)	
Computer	3.17 (1.305)	2.94 (1.311)	.30
Tablet	0.76 (1.306)	0.81 (1.283)	.97
Smartphone	3.81 (0.671)	3.48 (1.238)	.22
Game console	1.36 (1.376)	1.35 (1.391)	.93
Smart TV	0.88 (1.468)	1.44 (1.712)	.10

^a^Significance *P*<.05.

In terms of age (see Table 5), this study only found a slight statistically significant difference in the use of *smart TV*. Participants aged 30 years or above used *smart TV* less than younger adults. Although not statistically significant, a trend of greater use of most devices for the age range between 25 and 29 years can be observed across all participants. Nevertheless, it is worth highlighting that the age range in the last interval (≥30) is greater (30 to 53 years), which could affect the results.

For educational level (see Table 6), the only significant difference in terms of statistics is found for *computer*; the higher the educational level, the more frequent is the use. In general, participants with a high educational level used the analyzed devices more.

Table 7 shows some key details about the influence of the location where patients live. In this case, 2 different locations were analyzed, namely *rural* and *urban*. In general, the use of digital technology devices is greater in *urban* than in *rural* areas. There is, however, a statistically significant difference in the use of *game console*.

**Table 5:** Use of digital technology devices in patients with early-stage schizophrenia divided by age using the Kruskal-Wallis *H* test (N=90).

Digital device	Age (years)			*P* value^a^
	18-24 (37%), mean (SD)	25-29 (38%), mean (SD)	≥30 (25%), mean (SD)	
Computer	2.88 (1.386)	3.35 (0.884)	2.83 (1.642)	0.51
Tablet	0.67 (1.109)	0.97 (1.403)	0.70 (1.222)	0.53
Smartphone	3.58 (1.001)	3.82 (0.716)	3.43 (1.376)	0.26
Game console	1.18 (1.185)	1.74 (1.543)	1.04 (1.296)	0.16
Smart TV	1.30 (1.590)	1.53 (1.780)	0.48 (1.201)	0.03

^a^Significance *P*<.05*.*

**Table 6:** Use of digital technology devices in patients with early-stage schizophrenia divided by educational level using the Kruskal-Wallis *H* test (N=90).

Digital device	Educational level			*P* value^a^
	Basic (29%), mean (SD)	Medium (50%), mean (SD)	High (21%), mean (SD)	
Computer	2.50 (1.503)	3.04 (1.278)	3.79 (0.535)	.004
Tablet	0.77 (1.275)	0.62 (0.936)	1.21 (1.751)	.74
Smartphone	3.38 (1.299)	3.64 (1.026)	3.95 (0.230)	.14
Game console	1.23 (1.423)	1.24 (1.282)	1.79 (1.376)	.35
Smart TV	0.73 (1.218)	1.42 (1.764)	1.21 (1.686)	.37

^a^Significance *P*<.05.

**Table 7:** Use of digital technology devices in patients with early-stage schizophrenia divided by rural versus urban place of residence using the Mann-Whitney *U* test (N=90).

Digital device	Place of residence		*P* value^a^
	Rural (21%), mean (SD)	Urban (79%), mean (SD)	
Computer	3.11 (1.370)	3.03 (1.298)	.77
Tablet	0.47 (0.964)	0.87 (1.309)	.29
Smartphone	3.37 (1.499)	3.70 (0.852)	.93
Game console	0.89 (1.487)	1.48 (1.329)	.048
Smart TV	0.74 (1.522)	1.30 (1.634)	.08

^a^Significance *P*<.05.

**Table 8:** Comparison between patients with early-stage schizophrenia (N=90) and healthy participants (N=90) using the Mann-Whitney U test on the purpose of use of digital technology devices.

Participants		Patients, mean (SD)	Healthy participants, mean (SD)	*P* value^a^
**Entertainment**				
	Computer	0.76 (0.430)	0.71 (0.457)	>.99
	Tablet	0.74 (0.443)	0.80 (0.404)	.07
	Smartphone	0.82 (0.385)	0.91 (0.288)	.02
	Game console	0.74 (0.442)	0.95 (0.218)	.02
	Smart TV	0.82 (0.393)	0.92 (0.281)	.003
**Work**				
	Computer	0.63 (0.487)	0.81 (0.395)	.001
	Tablet	0.23 (0.426)	0.16 (0.373)	.19
	Smartphone	0.71 (0.454)	0.79 (0.412)	.10
	Game console	0.04 (0.191)	0.07 (0.250)	.41
	Smart TV	0.18 (0.393)	0.15 (0.363)	.60
**Socialization**				
	Computer	0.58 (0.566)	0.61 (0.491)	.27
	Tablet	0.23 (0.426)	0.20 (0.404)	.66
	Smartphone	0.88 (0.326)	0.98 (0.149)	.003
	Game console	0.52 (0.947)	0.25 (0.434)	.27
	Smart TV	0.18 (0.393)	0.08 (0.281)	.11
**Shopping**				
	Computer	0.47 (0.502)	0.69 (0.467)	.001
	Tablet	0.11 (0.323)	0.22 (0.417)	.34
	Smartphone	0.42 (0.496)	0.55 (0.500)	.05
	Game console	0.02 (0.136)	0.07 (0.250)	.17
	Smart TV	0.05 (0.226)	0.07 (0.254)	.71

^a^Significance *P*<.05.

### Purpose of Use of Digital Technology Devices

The purpose of use was classified into 4 domains: entertainment, work, socialization, and shopping.

We analyzed the responses of participants who reported using a specific device. Again, an ordinal variable was assigned to the responses with the following Boolean values: NO=0 and YES=1. This means that the answers of participants who reported never using a specific device were not considered.

Table 8 shows the results based on the number of patients and healthy individuals who used the devices: *computer* was used by 83 patients versus 89 healthy individuals, *tablet* by 35 patients versus 55 healthy individuals, *smartphone* by 84 patients versus 89 healthy individuals, *game console* by 54 patients versus 61 healthy individuals, and *smart TV* by 38 patients versus 59 healthy individuals.

As can be seen, *shopping* is the least used domain for all devices. The data show that patients do not frequently shop on the internet. Another relevant result is that the more versatile devices are *computer* and *smartphone*, whereas the others are mainly used for *entertainment*. Regarding *socialization*, the high value associated with *game console* is curious. This outcome may show that the games played by patients involve relevant social activity. As expected, the use of *smartphone* is also noteworthy in this domain.

However, are there any differences between patients and healthy participants in the purpose of use? Table 8 also shows the results of a case-control study using the Mann-Whitney *U* test for nonparametric variables.

Regarding *entertainment*, the mean of healthy participants is higher in general terms and there are significant differences in terms of statistics in the case of *smartphone*, *game console,* and *smart TV*, where healthy participants used these devices for *entertainment* more than patients.

In the case of *work*, *computer* was also used more by healthy participants. It is noticeable, although not from a statistical point of view, that *tablet* was used by patients more than by healthy participants. Nevertheless, the use of this technology was very low for both groups.

The purpose of *socialization* should be treated carefully. In fact, *smartphone* was used for *socialization* by a high number of patients and healthy participants, although healthy subjects prevail and there is statistical significance. However, on the contrary, *game console* was much more frequently used by patients than healthy participants.

For *shopping*, the difference in the use of *computer* is statistically significant in favor of healthy participants. Moreover, all other devices were found to be less used by patients for this purpose.

As noted before, both patients and healthy participants used most devices for the same purpose. Thus, the main purpose for using *smartphone* in both groups was *socialization*, followed by *entertainment*. Similarly, both *game console* and *smart TV* were used in both groups for the main purpose of *entertainment*. The main purpose of use of *tablet* was *entertainment* for both groups.

Regarding the purpose for using the *computer*, it is noticeable that patients used it preferentially for playing compared with healthy participants who primarily used it as a work tool.

### Use of the Internet

Regardless of device, what type of website do patients and healthy participants use when they access the internet? Do they use the internet to search for information or socialize? Participants were asked to indicate a maximum of 3 search engines.

Table 9 shows the outcomes regarding the search engines used by participants. The search engines were classified into *general* (general purpose), *entertainment*, *knowledge* (knowledge sharing), *shopping*, and *work*. As can be seen, the percentages are quite similar for patients and healthy participants.

As expected, a great majority of participants use *Google* as a search engine and *YouTube* for entertainment purposes and the use of specific websites for work and shopping is very low. For *work* and *shopping*, the specific search engines were not included, as almost every participant uses a different website. Moreover, the difference in the use of Wikipedia is interesting: 11.1% (10 out of 90 participants) for patients and 3.3% for healthy participants (3 out of 90 participants). When analyzing the number of answers provided by participants, only 5.6% (5 out of 90 participants) of patients did not indicate any search engine, whereas all healthy participants indicated at least one search engine. In addition, the percentage of participants indicating 3 apps was higher for healthy participants than patients.

Table 10 analyzes the apps used for socialization. As in the previous study, a classification was established to choose the main purpose of each app used, although it was difficult to categorize them into only one class.

### Use of Videogames

For videogames, Table 11 demonstrates that the participants were familiar with a great number of applications. Indeed, no single videogame was the most used by participants. The highest figures were for FIFA 17 in patients and for League of Legends in healthy participants.

**Table 9:** Use of search engines by patients with early-stage schizophrenia and healthy participants.

Participants		Patients		Healthy participants	
		Responses (N=163), n (%)	Individuals (N=90), n (%)	Responses (N=173), n (%)		Individuals (N=90), n (%)
**General**		96 (59)	—^a^	106 (61)		—
	Google	—	86 (96)	—		89 (99)
	Yahoo	—	9 (10)	—		9 (10)
	Bing	—	1 (1)	—		8 (9)
**Entertainment**		49 (30)	—	51 (29)		—
	YouTube	—	49 (54)	—		45 (50)
	Spotify	—	—	—		6 (7)
**Knowledge**		10 (6)	—	3 (2)		—
	Wikipedia	—	10 (11)	—		3 (3)
Shopping		5 (3)	5 (6)	6 (3)		6 (7)
Work		3 (2)	3 (3)	7 (4)		7 (8)

^a^Not applicable.

**Table 10:** App used for socialization by patients with early-stage schizophrenia and healthy participants.

Participants		Patients		Healthy participants	
		Responses (N=181), n (%)	Individuals (N=90), n (%)	Responses (N=223), n (%)	Individuals (N=90), n (%)
**Messaging**		95 (52)	—^a^	118 (539)	—
	WhatsApp	—	65 (72)	—	72 (80)
	Skype	—	12 (13)	—	9 (10)
	Twitter	—	9 (10)	—	33 (37)
	Snapchat	—	6 (7)	—	1 (1)
	WeChat	—	2 (2)	—	0 (0)
	Google Hangouts	—	1 (1)	—	0 (0)
	Telegram	—	0 (0)	—	3 (3)
**Social media**		53 (29)	—	65 (29)	—
	Facebook	—	53 (59)	—	65 (72)
**Photo sharing**		28 (15)	—	35 (16)	—
	Instagram	—	28 (31)	—	35 (39)
Business and employment		3 (2)	—	3 (1)	6 (7)
Catalog of ideas		1 (1)	—	1 (0)	7 (8)
Dating		1 (1)	—	1 (0)	7 (8)

^a^Not applicable.

**Table 11:** Use of videogames in patients with early-stage schizophrenia versus a control sample.

Videogames	Patients (N=75), n (%)	Healthy participants (N=95), n (%)
FIFA 17	12 (16)	7 (7)
League of Legends	5 (7)	10 (10)
Overwatch	2 (3)	8 (8)
Mario Kart 8	7 (9)	1 (1)
Grand Theft Auto V	6 (8)	1 (1)
The Elder Scrolls V: Skyrim	0 (0)	6 (6)
Call of Duty: Infinite Warfare	5 (7)	3 (3)
The Legend of Zelda: Breath of the Wild	1 (1)	5 (5)
Minecraft	4 (5)	2 (2)
Pokémon GO	0 (0)	4 (4)
Hearthstone: Heroes of Warcraft	0 (0)	3 (3)
The Sims 4	1 (1)	3 (3)
Others	36 (48)	42 (44)

## Discussion

### Principal Findings

To our knowledge, this is the first multicentric study on the use of digital technology for internet access in patients with early-stage schizophrenia compared with a sample of control participants matched in age, gender, and place of residence. The smartphone is clearly the digital device most widely used by patients (84 out of 90 participants), reaching almost 95% of use at least twice a week. The second most used device is the computer, which reaches a use of almost 75% (67 out of 90 participants) at least twice per week. With regard to the other devices, the low usage indicated and the high number of *never, but would like to* responses is striking. This might be because these devices are not economically affordable for this group of the Spanish population despite there being outstanding interest in them.

In any event, although the use of all devices is lower in patients compared with healthy participants, the results indicate that a large proportion of patients with early-stage schizophrenia have access to different technological devices and use them frequently. This finding is of great importance for designing intervention programs including the use of technological devices, especially smartphones and computers. The inclusion of technological devices is being investigated in several areas such as neurocognitive remediation [34], adherence to pharmacological treatment [35], social cognition remediation [36], treatment of refractory auditory hallucinations [37], or training in social skills [38].

Moreover, no statistically significant differences were found in the *frequency of* use of digital technology devices between male and female patients, which is interesting in terms of gender equality. In addition, no statistically significant differences were detected in terms of age, except for a lower use of smart TV in older adults. It was also found that frequency of use of computers increased significantly as educational level increased. Although not statistically significant, patients who lived in cities used most digital devices more frequently than those who lived in small towns. To sum up, healthy participants used all digital technology devices more frequently than patients, with this difference between the groups being statistically significant for all devices except for game console.

The main results in terms of *purpose of use of digital technology* when accessing the internet are described below. Patients and healthy participants coincided in that the main purpose of use for tablet, game console, and smart TV was entertainment and for smartphone, socialization. However, they differ in the purpose for using the computer, with entertainment being the principal motivation for patients and work for healthy participants. In general, most devices are less used by patients for this purpose. These data could indicate a lower interest of patients for this purpose.

Specifically, entertainment seems to be the main objective sought with most devices, both in patients and in healthy participants. For entertainment, patients use smart TV and smartphone more frequently, whereas healthy participants prefer the game console, with this difference being statistically significant. For work, there is a statistically significant difference in favor of healthy participants with respect to computer use. The devices most used to socialize are smartphone (with a statistically significant difference in favor of healthy participants) and computer in both groups. Another finding is that patients used the game console more frequently than healthy participants for socialization purposes. Web-based shopping seems to be the least common objective in both groups. Nevertheless, healthy participants engaged more in this type of activity, especially on the computer.

With regard to the most used *search tools*, the main finding is the lack of statistically significant differences between the 2 groups. Google is the most important search engine followed by YouTube. The access to social networks is quite similar in both groups, with WhatsApp and Facebook being the most important applications. As expected, the most frequently used domain of social activity was messaging, followed by social media and photo sharing. However, the use of Twitter, another well-known app in this domain, is slightly curious. Here, patients presented a lower use of this app than healthy participants, which was nevertheless mitigated through other messaging apps such as Snapchat and WeChat.

Regarding the number of answers provided by the participants, it is surprising that several patients with early-stage schizophrenia indicated no app, whereas this percentage was null for the other group. Moreover, the percentage of participants reporting a maximum of 3 apps was higher in the case of healthy participants.

In the case of *videogames*, both groups knew and played several video games, with FIFA 17 being the favorite for patients and League of Legends for healthy participants. Nonetheless, no single videogame was used by most participants. This means that an accurate analysis cannot be performed by just looking at the results provided in this table. Therefore, a deeper analysis, probably mining the most important features of the videogames, is necessary to give any concluding remarks in this regard.

### Strengths and Limitations

There are several strengths and limitations in this study.

The main strength of this work is that it is the first multicentric study carried out on internet access through digital technology (with this degree of depth in terms of frequency of use, purpose of use, and type of digital technology) by patients with early-stage schizophrenia in Spain compared with a sample of controls matched in age, gender, and place of residence.

It should also be highlighted that the selection of a multicentric approach representing 3 typical, albeit different, cities is essential to generalize the results to the overall Spanish population. Indeed, this research study involved participants from 3 different recruitment centers (cities): *Hospital Virgen de la Luz* (Cuenca), *Hospital Universitario de San Juan* (Alicante), and *Hospital Universitario 12 de Octubre* (Madrid). The 3 centers of recruitment are related to 3 different types of settlements based on their number of inhabitants. The first could be classified as a *large town*, the second a *large city*, and the last a *metropolis*. Moreover, in terms of rural population, Cuenca has a high percentage, in Alicante the percentage is small, and in Madrid it is practically null.

In addition, regarding the statistical analysis, the size of the sample (180 participants) can be considered more than enough to extract important and useful considerations. In this sense, another strength of the study is having a control group of the same sociocultural environment, matched in age and gender.

There are some limitations to our study, mainly that socioeconomic status, ethnicity, culture, and working status of participants were not collected, which would have helped enhance the explanation of some results obtained.

### Comparison With Prior Works

To the best of our knowledge, no other published multicentric studies have analyzed the access and use of digital technology devices and applications in patients with early-stage schizophrenia in comparison with a sample of healthy participants matched in age, gender, and place of residence.

The results of this study indicate that a large proportion of patients have access to different technological devices and use them frequently, although the use of all devices is lower in patients compared with healthy participants. This lower use could be attributed to lower economical level of the patients [39], lower academic level [40], their cognitive dysfunction [41], unemployment rates [42], or the presence of negative symptoms [43]. Our results for access to different technological devices and use coincide with the results of a previous study, which concluded that more than 90% of the psychotic patients (not only the ones in early stage) surveyed owned more than one digital device and most of them used multiple devices habitually [12].

Both patients and healthy participants coincide in the preference for using smartphone over the other devices. The second most used device is the computer, followed by smart TV, game console, and tablet. The preference for the use of smartphone conforms to the Spanish population’s access to this device, as well as the preference of its use to access the internet [1]. Considering this previous report, the smartphone seems to be the most used device by the Spanish population, both for healthy participants and patients, which coincides with the results obtained in our own study.

A recent meta-analysis on smartphone ownership in psychotic patients revealed that it was increasing rapidly, with 81.4% ownership among respondents between 2014 and 2015 in the United States [44]. Another study carried out in Georgia in 2015, with a sample of 80 patients with schizophrenia, concluded that there was greater access to smartphones compared with computers in the study sample (73% vs 54%), which would also be compatible with our results [24].

In addition, in a previous survey of access and use of technological tools carried out in 2012 in Montreal on 71 patients with a first psychotic episode [29], a greater use of computer (desktop or laptop) was reported in comparison with mobile phone or smartphone, reaching 96% versus 70% of frequency (defined as *from daily use up to 2 to 3 times per week*). This difference in the preference for computer compared with smartphone could be related to 2 circumstances. The first could be cultural difference, as in 2017, the device most frequently used to access the internet was smartphone (76%), followed closely by laptop computer (71%), according to the National Statistics Institute of Canada [45]. As can be concluded from these results, the Canadian population has no marked preference for the smartphone, with both having practically the same frequency of use. The second reason could be due to the fact that the study was carried out in 2012 and the pattern of use of digital technology is constantly changing and adapting to the needs of the population. Unlike other previously published studies, a greater use of game consoles in men was not observed [29].

Some years ago, an interesting study found that the internet is an influential source of illness-related information for patients with schizophrenia [46]. Moreover, the same report stated that many aspects of their behavior related to internet usage resembled those of individuals without mental illness. The following paragraphs support this statement.

Regarding the most visited search engines, the responses of both groups (patients and healthy participants) were quite similar. Google is the most named search engine by far (with close to 100% use in both groups), which shows the enormous expansion of this tool. The results (although not as overwhelming) were similar for YouTube, the main page visited by both groups for entertainment. It is striking that both patients and healthy participants named these search tools freely, that is, without a list provided by the interviewer. At the time of data collection, both groups coincided in the use of Wikipedia, the use being higher in patients than in healthy participants. With regard to other types of websites related to job searching, the response rate was low in both groups, although higher in the healthy participants, which could be related to the lower employment rates in the psychotic population.

With regard to the applications used for socialization, it is striking that around 12% of patients use no application. This is probably related to the difficulties these patients present when socializing. Nevertheless, the patients who responded did so similarly to the healthy participants, with WhatsApp being the favorite app, followed by Facebook and Instagram. As with Google and YouTube, it can be concluded that these applications have expanded enormously in recent years, reaching even young psychotic patients. Both groups agree on the low rate of responses in the use of dating websites, which could be related to inhibition when verbalizing this type of information.

### Conclusions

This is the first multicentric study carried out that provides information about the access to the internet and use of digital technology devices and applications by patients with early-stage schizophrenia in comparison with healthy participants matched in age, gender, and place of residence.

In general terms, the results obtained in our study indicated that a large proportion of patients with early-stage schizophrenia have access to different digital devices and use them frequently. However, a lower frequency of use of most devices was found in patients compared with healthy participants. For some devices, this was due to a lack of access, not an absence of interest. Nevertheless, both groups coincide in the most used devices. In addition, the purpose of using the devices in relation to the internet is highly similar in both groups.

This study brings the scientific community closer to the patterns of internet access and use of digital technology in patients with early-stage schizophrenia compared with healthy participants from the same demographic areas. The analysis of this information will be useful to guide the future development of internet technology–based therapeutic applications [47].

## Appendix

Multimedia Appendix 1 Consolidated criteria for reporting qualitative studies (COREQ): 32-item checklist.

## Abbreviations

eHealth: electronic health
DSM-5: Diagnostic and Statistical Manual of Mental Disorders, fifth edition
INE: Instituto Nacional de Estadística
ITU: International Telecommunication Union
mHealth: mobile health
