# Supplementary material for: Digital Technology for Internet Access by Patients With Early-Stage Schizophrenia in Spain: Multicenter Research Study
Source: J Med Internet Res. 2019 Apr 5;21(4):e11824. doi: 10.2196/11824 (PMC6533031; doi:10.2196/11824)
Supplement: Multimedia Appendix 1 [file jmir_v21i4e11824_app1.docx]

# Multimedia Appendix 1.

# Consolidated criteria for reporting qualitative studies (COREQ): 32-item checklist

##

| **No.** | **Item** | **Guide question** | **Response** |
| --- | --- | --- | --- |
| **Domain 1: Research team and reflexivity** | | | |
| Personal characteristics | | | |
| 1. | Interviewer/facilitator | Which author/s conducted the interview or focus group? | Patricia Fernández-Sotos carried out the interview in Madrid, Ana Isabel Aparicio in Cuenca and Lorena García-Fernández in San Juan, Alicante. |
| 2. | Credentials | What were the researcher’s credentials? *E.g. PhD, MD* | Please see affiliations. |
| 3. | Occupation | What was their occupation at the time of the study? | Please see affiliations. |
| 4. | Gender | Was the researcher male or female? | 6 females and 4 males. |
| 5. | Experience and training | What experience or training did the researcher have? | The researchers have years of experience in clinical research with patients with schizophrenia. |
| Relationship with participants | | | |
| 6. | Relationship established | Was a relationship established prior to study commencement? | Patients were known in advance by the interview group. No previous relationship was established with healthy people. |
| 7. | Participant knowledge of the interviewer | What did the participants know about the researcher? *e.g. personal goals, reasons for doing the research* | The participants knew that the interviewers were part of the clinical team of each center. They knew that our clinical groups are also research groups, with a common objective of improving the knowledge about schizophrenia disorder in order to improve the functioning of patients in real life. |
| 8. | Interviewer characteristics | What characteristics were reported about the interviewer/facilitator? *e.g. Bias, assumptions, reasons and interests in the research topic* | The interviewers provided information in this regard, especially about the interest in knowing the use and patterns of Internet access in patients compared to healthy participants. |

| **Domain 2: Study design** | | | |
| --- | --- | --- | --- |
| Theoretical framework | | | |
| 9. | Methodological orientation and Theory | What methodological orientation was stated to underpin the study? *e.g. grounded theory, discourse analysis, ethnography, phenomenology, content analysis* | Grounded theory with one data collection episode in form of a semi-structured interview. |
| Participant selection | | | |
| 10. | Sampling | How were participants selected? *e.g. purposive, convenience, consecutive, snowball* | Consecutive purposive sampling for patients and convenience sampling for healthy participants. |
| 11. | Method of approach | How were participants approached? *e.g. face-to-face, telephone, mail, email* | Face-to-face approach. |
| 12. | Sample size | How many participants were in the study? | 90 patients and 90 healthy participants. |
| 13. | Non-participation | How many people refused to participate or dropped out? Reasons? | 10 patients and 23 healthy people. The main reason for not participating was that they did not have time or had no interest in the study. |
| Setting | | | |
| 14. | Setting of data collection | Where was the data collected? *e.g. home, clinic, workplace* | At clinic, the usual workplace of the interviewers. |
| 15. | Presence of non-participants | Was anyone else present besides the participants and researchers? | No, the interviewer and the participant were alone during the interview. |
| 16. | Description of sample | What are the important characteristics of the sample? *e.g. demographic data, date* | \|  \| **Patients**  **(n=90)** \| **Healthy participants (n=90)** \| **Statistics ^(*)^ and significance** \| \| --- \| --- \| --- \| --- \| \| **Age** \| 28.1 ± 8.6 years \| 27.9 ± 8.7 years \| t=0.189  p=0.850 \| \| **Gender** \| Men: 53.3 %  Women: 46.7 % \| Men: 56.7 %  Women: 43.3 % \| *X*^2^=0.202  p=0.653 \| \| **Educational level** \| Basic: 28.9 %  Medium: 50.0 %  High: 21.1 % \| Basic: 22.2 %  Medium: 36.7 %  High: 41.1 % \| *X*^2^=8.414  p=0.015 \| \| ^*^ t: Student’s t  *X*^2^: chi-square \| \| \| \| |
| Data collection | | | |
| 17. | Interview guide | Were questions, prompts, guides provided by the authors? Was it pilot tested? | The authors provided guides. A pilot study was carried out in the coordinator center (Madrid) with 5 patients and 5 healthy participants. |
| 18. | Repeat interviews | Were repeat interviews carried out? If yes, how many? | No, each participant was only interviewed one. |
| 19. | Audio/visual recording | Did the research use audio or visual recording to collect the data? | No, only a semi-structured questionnaire was filled out. |
| 20. | Field notes | Were field notes made during and/or after the interview or focus group? | Yes, field notes were made during the interviews. |
| 21. | Duration | What was the duration of the interviews or focus group? | Average duration of 20-30 minutes. |
| 22. | Data saturation | Was data saturation discussed? | No. |
| 23. | Transcripts returned | Were transcripts returned to participants for comment and/or correction? | At the end of the interview, data were revised with each participant. |

| **Domain 3: Analysis and findings** | | | |
| --- | --- | --- | --- |
| Data analysis | | | |
| 24. | Number of data coders | How many data coders coded the data? | Three. |
| 25. | Description of the coding tree | Did authors provide a description of the coding tree? | Yes. |
| 26. | Derivation of themes | Were themes identified in advance or derived from the data? | Themes were identified in advance. |
| 27. | Software | What software, if applicable, was used to manage the data? | The Statistical Package for the Social Sciences (SPSS) version 23.0 (IBM Corp) was employed for the statistical analysis of all usable data. |
| 28. | Participant checking | Did participants provide feedback on the findings? | We will offer it when they were published. |
| Reporting | | | |
| 29. | Quotations presented | Were participant quotations presented to illustrate the themes / findings? Was each quotation identified? *e.g. participant number* | No. It was not necessary. |
| 30. | Data and findings consistent | Was there consistency between the data presented and the findings? | Yes. |
| 31. | Clarity of major themes | Were major themes clearly presented in the findings? | Yes. |
| 32. | Clarity of minor themes | Is there a description of diverse cases or discussion of minor themes? | Yes. |
